# Time and spatially resolved tracking of the air quality in local public transport

**DOI:** 10.1038/s41598-022-07290-5

**Published:** 2022-02-28

**Authors:** Tunga Salthammer, Christian Fauck, Alexander Omelan, Sebastian Wientzek, Erik Uhde

**Affiliations:** Department of Material Analysis and Indoor Chemistry, Fraunhofer WKI, Bienroder Weg 54 E, 38108 Brunswick, Germany

**Keywords:** Environmental sciences, Environmental monitoring, Sensors

## Abstract

As an indoor environment, public transport is subject to special conditions with many passengers in a comparatively small space. Therefore, both an efficient control of the climatic parameters and a good air exchange are necessary to avoid transmission and spread of respiratory diseases. However, in such a dynamic system it is practically impossible to determine pathogenic substances with the necessary temporal and spatial resolution, but easy-to-measure parameters allow the air quality to be assessed in a passenger compartment. Carbon dioxide has already proven to be a useful indicator, especially in environments with a high occupancy of people. Airborne particulate matter can also be an important aspect for assessing the air quality in an indoor space. Consequently, the time courses of temperature, relative humidity, carbon dioxide and particulate matter (PM_10_) were tracked and evaluated in local public transport buses, trams and trains in the Brunswick/Hanover region. In all measurements, the climatic conditions were comfortable for the passengers. Carbon dioxide was strongly correlated with occupancy and has proven to be the most informative parameter. The PM_10_ concentration, however, often correlated with the dynamics of people when getting on and off, but not with the occupancy. Sensors, equipped with integrated GPS, were installed in the passenger cabins and were found to be useful for recording location-related effects such as stops. The results of this study show that the online recording of simple parameters is a valuable tool for assessing air quality as a function of time, location and number of people. When the occupancy is high, a low carbon dioxide level indicates good ventilation, which automatically reduces the risk of infection. It is therefore recommended to take more advantage of low-cost sensors as a control for air conditioning systems in passenger cabins and for evaluations of the dynamics in public transport.

## Introduction

The rapid, worldwide spread of SARS-CoV-2 viruses is closely linked to the fast, convenient and advanced national and international transport systems. There are many reports suggesting that the transport sector contributes to the transmission and infection chains of respiratory diseases^1^, e.g. in buses^2–5^, in rail transport^6–10^, on cruise ships^11^ and in aircraft cabins^12–17^.

The transmission of pathogenic germs can take place via various routes. Against the classic droplet infection that occurs over short distances, maintaining a spatial distance is a sensible and efficient measure. Regular disinfection of skin and material surfaces should limit or avoid infection through direct contact. However, it is convincingly argued that many types of pathogens such as the SARS-CoV-2 virus could be transported over longer distances as components of aerosols in the air^18–20^. Therefore, appropriate control and protective measures should also be taken into account against this path of infection.

The public transport system is subject to special climatic conditions with a large number of passengers in a confined space. Browne et al.^21^ published an overview of the transmission of influenza and coronaviruses during passenger transport and in traffic hubs, in which 41 studies from the areas of air transport, sea transport and ground transport were taken into account. With regard to the air transmission path, the highest possible air exchange rate with a maximum proportion of fresh air should be aimed for in order to reduce risk. Intensive mixing of the air is avoided by means of displacement ventilation, temperature and relative humidity also influence the survival of viruses in the environment^22–26^.

Online measurement of airborne pathogens with the necessary time resolution in a dynamic system such as local passenger traffic is practically impossible. However, other parameters that are easy to measure are available that allow the assessment of the ventilation efficiency in a passenger compartment. In particular, the carbon dioxide (CO_2_) concentration can serve as a suitable indicator in spaces with a high density of people^27^. The determination of the particle concentration, especially PM_10_, is also an important aspect for evaluating the air quality in an indoor space^28,29^. Boniardi et al.^30^ measured multiple air pollutants to compare exposure when commuting by car, public transport and bike.

In this work the results of measurements for climatic parameters (temperature and relative humidity), carbon dioxide (CO_2_) and particles (PM_10_), carried out in public transport areas in the area of Brunswick/Hanover, Germany, are presented and discussed. In the course of the project, space-saving sensors were developed with which the air quality in the passenger areas can not only be monitored online efficiently, but also record location-related effects such as stops and train stations using integrated GPS.

## Methodology

### Measuring devices

Hand-held devices (Rotronic Messgeräte GmbH, Model CP11) were used to measure temperature T (°C), relative humidity RH (%) and CO_2_ (ppm). The time resolution is 10 s. The measuring ranges are − 20 °C to + 60 °C (temperature), 0.1% to 99.95% (relative humidity) and 0 ppm to 5000 ppm (CO_2_). The measuring principle for CO_2_ is non-dispersive infrared (NDIR). An Optical Particle Sizer (TSI Inc. Model 3330) was used to measure the particle concentration. The measuring range is 0.3 µm to 10 µm with a size resolution of 16 channels. The time resolution is 20 s. The device measures the particle number concentration by means of light scattering. The conversion to mass-related concentrations is based on the assumption of spherical particles and a density of 1 g/cm^3^. The measurement of PM_2.5_ and PM_10_ is particularly important for exposure and health-related studies. Since this is only about the dynamics of particles, PM_10_ is used as the measurement parameter. PM_10_ refers to the mass-related sum of particles with an aerodynamic diameter < 10 µm, whereby a separation efficiency of 50% is assumed for the 10 µm fraction^31^. Moreover, the high fluctuations in the concentrations of the smaller particle fractions did not allow any conclusions to be drawn. The results described here were obtained in the middle of the passenger cabins. Sampling took place at a height of 1.5 m. Further measurements were carried out near the entry and exit doors, but the results were not significantly different. According to the operators, the ventilation systems were set for high performance and a high proportion of fresh air. This indicates a quick and even distribution of the air constituents.

For the background concentrations of PM_10_, data were retrieved from the following stations of the Lower Saxony Air Monitoring Network (LÜN): DENI011 (Brunswick, urban background) and DENI054 (Hanover, urban background).

The data were analyzed with OriginPro, Version 2021b (OriginLab Corporation, Northhampton, MA).

### Sensors

A portable, battery driven monitoring system was also used for the recording of environmental parameters. The system consisted of a SCD30 NDIR CO_2_ sensor, a SPS30 optical particle sensor (both Sensirion AG, Staefa, Switzerland), a BME280 temperature/humidity/pressure sensor (Bosch Sensortec GmbH, Reutlingen, Germany), and a SAM-M8Q GNSS antenna module (U-Bloxx, Thalwil, Switzerland). The measurements and the position data were logged in 11 s intervals to a SD memory card using a SAMD21 microcontroller (Microchip Technology Inc., Chandler, USA).

The particle sensor measures PM_1_, PM_2.5_, and PM_10_ with a resolution of 1 µg/m^3^, but also outputs particle number concentrations for the respective size bins. The CO_2_ sensor has a resolution of 30 ppm CO_2_ and is corrected for temperature deviations by an onboard temperature sensor. The temperature/pressure/humidity sensor has an accuracy of ± 0.5 °C, ± 2 hPa and ± 3% RH, respectively.

### Measuring program

The research program focused on regional traffic in Brunswick (Braunschweig) and Hanover, or between the two cities. All measurements were carried out between December 2020 and March 2021, always from the first to the last station. This was done in consultation and with the approval of the respective operator, Braunschweiger Verkehrs GmbH (BSVG), WestfalenBahn GmbH (WfB), RegioBus GmbH and ÜSTRA Hannoversche Verkehrsbetriebe. All lines considered are summarized in Table 1. The exact type designations of the vehicles, PM_10_ concentrations in the ambient air, in the Brunswick and Hanover Central metro station as well as route maps are shown in the Supporting Information. The occupancy of the passenger area was recorded for all route sections. Since the respective vehicle stops every minute in city traffic, orders of magnitude were specified, which the measuring staff had to indicate by ticking the corresponding stops. Seats: (a) not or little occupied (< 10%); (b) weakly occupied (10–30%); (c) moderately occupied (30–70%); (d) crowded (> 70%). Standing places: (a) not or little occupied; (b) weakly occupied; (c) crowded. For each measurement, the occupancy was recorded by two staff persons who were in the same area but at different locations (in the front section of the cabin and near a door in the middle). In addition, a measurement was carried out on the platform of the ÜSTRA Tram 4 underground station at Hanover Central.

**Table 1:** Compilation of all measurements carried out in regional traffic between Brunswick and Hanover.

Run	Date	Time	Operator	Line	Route
01	04.12.2020	07:01–08:02	BSVG	Bus Line 411	Lammer Busch–Am Kalkwerk
02	04.12.2020	08:09–09:09	BSVG	Bus Line 411	Am Kalkwerk–Lammer Busch
03	08.12.2020	07:01–07:37	BSVG	Tram Line 3	Weserstr.–Grenzweg
04	08.12.2020	07:40–08:15	BSVG	Tram Line 3	Grenzweg–Weserstr
05	01.03.2021	15:45–16:16	BSVG	Tram Line 10	Central Station–Carl-Miele-Str
06	01.03.2021	16:25–16:54	BSVG	Tram Line 10	Carl-Miele-Str.–Central Station
07	01.03.2021	17:00–17:27	BSVG	Tram Line 10	Central Station–Carl-Miele-Str
08	01.03.2021	17:40–18:06	BSVG	Tram Line 10	Carl-Miele-Str.–Central Station
09	04.03.2021	15:47–16:26	BSVG	Bus Line 419	Circle Line Central Station
10	04.03.2012	16:47–17:24	BSVG	Bus Line 419	Circle Line Central Station
11	04.03.2021	17:46–18:24	BSVG	Bus Line 419	Circle Line Central Station
12	14.12.2020	06:20–07:03	WfB	95772	Brunswick–Hanover
13	14.12.2020	07:16–08:06	WfB	95753	Hanover–Brunswick
14	14.12.2020	16:20–17:05	WfB	95782	Brunswick–Hanover
15	14.12.2020	17:16–18:02	WfB	95811	Hanover–Brunswick
16	09.03.2021	07:30–08:13	ÜSTRA	Tram Line 4	Roderbruch–Garbsen
17	09.03.2021	08:30–09:14	ÜSTRA	Tram Line 4	Garbsen–Roderbruch
18	09.03.2021	14:50–15:34	ÜSTRA	Tram Line 4	Roderbruch–Garbsen
19	09.03.2021	15:50–16:34	ÜSTRA	Tram Line 4	Garbsen–Roderbruch
20	09.03.2021	10:32–11:20	RegioBus	Regiobus 500	Hanover Bus Station–Gehrden
21	09.03.2021	11:39–12:31	RegioBus	Regiobus 500	Gehrden–Hanover Bus Station

BSVG: measurements in Brunswick; WfB: measurements between Brunswick and Hanover; ÜSTRA: measurements in Hanover; RegioBus: measurements in Hanover region. Route maps are shown in the Supporting Information.

## Results

In coordination with the operator, routes and times were selected in such a way that, under the given circumstances, a high occupancy rate could be expected, either in the early morning or in the afternoon (see Table 1).

**Figure 1:**
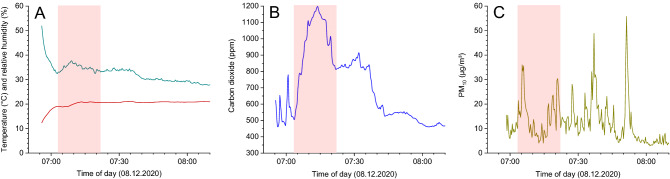
Time curves for temperature and relative humidity (**A**), carbon dioxide concentration (**B**) and PM_10_ concentration (**C**) for BSVG Line 3 (Run 03). The red area marks periods with high occupancy (> 70%).

**Figure 2:**
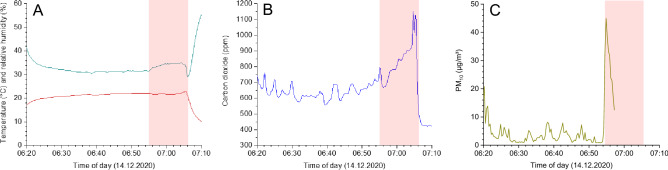
Time curves for temperature and relative humidity (**A**), carbon dioxide concentration (**B**) and PM_10_ concentration (**C**) for WestfalenBahn Line 95772 (Run 12). The red area marks periods with high occupancy (> 70%).

The large data sets and the sometimes high fluctuations make a comparative presentation of all results difficult. For CO_2_ no mean values were calculated for the respective route, as the values fluctuated strongly depending on occupancy. The maximum values are therefore provided for each measurement run in Table 2 and compared with hygiene-based guide values. Maximum values are also presented for temperature and relative humidity with the relative humidity relating to the corresponding temperature. In case of PM_10_, maximum values and arithmetic means are shown in Table 2. The respective time courses plotted in Figs. 1 and 2 are representative of all measurements.

**Table 2:** Results of the measurements in buses, trams and trains in public transport in the Brunswick/Hanover area.

Run	Operator	Line	Maximum				Mean
			T (°C)	RH (%, at max T)	CO_2_^a^ (ppm)	PM_10_^b^ (µg/m^3^)	PM_10_ (µg/m^3^)
01	BSVG	Bus Line 411	23.8	19	960	67.1	12.9
02	BSVG	Bus Line 411	22.3	19	780	26.8	8.7
03	BSVG	Tram Line 3	21.1	39	1197	48.9	15.2
04	BSVG	Tram Line 3	20.9	30	605	55.8	8.7
05	BSVG	Tram Line 10	21.2	29	851	49.7	19.3
06	BSVG	Tram Line 10	22.3	31	776	44.0	16.5
07	BSVG	Tram Line 10	21.1	32	666	76.7	18.7
08	BSVG	Tram Line 10	20.1	36	821	62.0	17.1
09	BSVG	Bus Line 419	21.4	34	995	101.4	41.1
10	BSVG	Bus Line 419	22.7	29	913	63.5	32.5
11	BSVG	Bus Line 419	22.2	30	897	52.8	24.3
12	WfB	95772	22.7	29	1151	45.0	6.1
13	WfB	95753	21.2	29	790	25.5	3.3
14	WfB	95782	21.7	38	1105	30.1	5.0
15	WfB	95811	23.0	32	666	42.0	6.2
16	ÜSTRA	Tram Line 4	19.6	40	981	73.1	13.2
17	ÜSTRA	Tram Line 4	18.1	43	754	28.9	9.2
18	ÜSTRA	Tram Line 4	19.3	39	923	34.9	7.0
19	ÜSTRA	Tram Line 4	18.6	40	762	68.2	11.7
20	RegioBus	Regiobus 500	20.9	33	1047	83.1	8.4
21	RegioBus	Regiobus 500	22.8	33	1161	10.3	3.8

The maximum values are given for temperature, relative humidity and carbon dioxide. For PM_10_ maxima and arithmetic means are provided. Larger deviations from the maximum values were not observed for T and RH. For carbon dioxide and PM_10_, the respective background values corresponded to the ambient air concentration.

^a^The minimum corresponded to the ambient air concentration of approx. 400 ppm.

^b^The minimum corresponded to the ambient air concentration of approx. 3–10 µg/m^3^.

## Discussion

### Temperature and relative humidity

During all measurement runs, the temperature in the passenger cabins was very constant and, with values between 18 and 24 °C, in a comfortable range for the passengers. In Figs. 1A and 2A examples for the BSVG Tram 3 (Run 03) and the WestfalenBahn (Run 12) are shown. Significant deviations from the maximum temperature given in Table 2 were not observed. This also applies to periods more than 70% total occupancy (areas marked in red). The lower temperatures or high humidity at the beginning of the measurement can be assigned to influences of ambient air. With the exception of Runs 01 and 02, each with 19% RH, the relative humidity was in the range of approx. 30% to 40%, which can also be rated as pleasant. With regard to the indoor climate, humans are not very adaptable, because even small temperature fluctuations and air movements can cause thermal discomfort. In particular, the sensation of warmth depends on the physical activity, clothing, air temperature, mean radiation temperature, air speed and humidity. At 20 °C, people find a relative humidity of 40% to 60% pleasant^32^. In buses and trains, 20–22 °C are usually the target values, but drafts are unavoidable due to regular opening of windows and doors. However, the very constant values show that the air conditioning systems of the transport vehicles have effective control technology. No changes could be recorded while the doors were being opened at the bus stops. The outside air temperature on the measurement days was less than 10 °C. When there was a high density of people, a slight but significant increase in the relative humidity was observed.

### Carbon dioxide

With regard to hygienic aspects, the carbon dioxide concentration in the room air serves as an indicator for air deterioration caused by human activity. The concept of the German Committee on Indoor Guide Values regards CO_2_ concentrations of less than 1000 ppm (0.1 vol%) as hygienically harmless and CO_2_ concentrations higher than 2000 ppm as hygienically unacceptable^33^. In rooms with a high density of people like school classrooms, the carbon dioxide concentration can serve as a guide for good or bad ventilation^27^. Carbon dioxide has also long been regarded as a reliable indicator for the air exchange rate. A CO_2_ concentration of at most 1000 ppm indicates a hygienically adequate air exchange under normal living conditions. This means that information can be provided quickly and easily as to whether and when ventilation is necessary. However, this does not mean that a CO_2_ concentration of less than 1000 ppm generally protects against infection. On the other hand, CO_2_ concentrations significantly or permanently higher than 1000 ppm indicate inadequate ventilation management with a potentially increased risk of infection.

During light physical activity, an adult emits approximately 18–24 l/h CO_2_^34^. The ambient air concentration of CO_2_ in urban areas is approximately 400 ppm. A standard articulated bus has an internal volume of around 120 m^3^. In order to keep the CO_2_ concentration permanently below 1000 ppm with an occupancy of 50 people and an emission rate of 20 l/h, an air exchange of at least 15 h^−1^ would be necessary (see Supporting Information for details). It can thus be estimated that the fresh air supply must be at least 10 l /s /p (liters per second and person).

As can be seen from Figs. 1B and 2B, the carbon dioxide concentration correlates, as expected, with the density of people in the passenger cabin. Figure 1B represents Run 03 in the BSVG Tram 3 in the early morning. In the section marked in red there was a large number of students on the tram (occupancy > 70%) and the carbon dioxide concentration reached a maximum value of 1197 ppm. The measurement shown in Fig. 2B was also carried out in the early morning on the WestfalenBahn (Run 12). From Lehrte, the last stop before Hanover Central, the train was heavily frequented by commuter traffic, which led to a CO_2_ concentration of 1151 ppm. However, these values are only slightly above the guide value for harmless concentrations of 1000 ppm, and far below the value of 2000 ppm, from which unacceptable conditions prevail. The other measurements led to similar results (see Table 2). It can therefore be concluded that the ventilation systems in the passenger cabins work efficiently. The ambient air concentrations of CO_2_ were in all cases in the range of 400 ppm.

Kwon et al.^35^ carried out measurements in the metropolitan subway of Seoul, Korea and found CO_2_ peak concentrations of more than 4000 ppm during the rush hour. An extensive literature study on CO_2_ in passenger cabins of buses and trains was presented by Querol et al.^17^ In many cases CO_2_ concentrations in the range of 2000–4000 ppm have been reported. Querol et al.^17^ see evidence for a lack of ventilation and consequently an increased risk of aerosol COVID-19 transmission. In the same publication, Querol et al.^17^ also present the results of CO_2_ measurements from 37 public bus trips during rush hour in Barcelona. In this context, scenarios with insufficient ventilation were also examined, which leads to maximum CO_2_ contractions of approximately 1800 ppm. Otherwise, despite the different transport systems and metropolises, our CO_2_ concentration courses were definitely comparable with their data.

### Particulate matter

**Figure 3:**
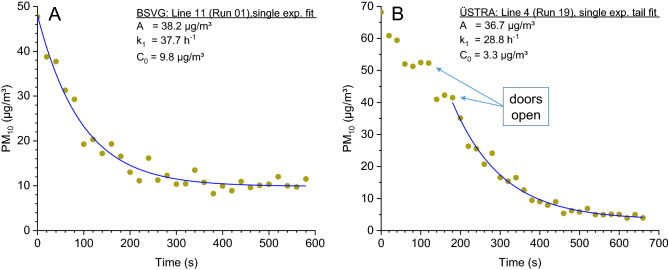
Time course of the PM_10_ concentration and single exponential non-linear regression analysis with Eq. (1). (**A**) BSVG Line 411 (Run 01); (**B**) ÜSTRA Line 4 (Run 19). See Figure S1 in the Supporting Information for the full-time course of PM_10_ concentrations.

In the case of the particles, the concentration peaks do not necessarily correspond to the occupancy density of the passengers, but often to their dynamics when the doors are regularly opened, combined with getting on and off. Some of the particles are brought in through the ambient air, some through clothing and the movement of passengers. As a result, the particle concentration in the cabin increases significantly at the stations. It was also noticeable that in various measurements the highest particle concentrations were recorded when several passengers boarded at the same time at the starting point. It is known that moving people are relevant particle sources or resuspend particles^36^, but it is certain that the passengers' breathing is not the source of the particles. The maxima occur only for a short time and reach the base level again within a few seconds (see Fig. 3). For each measurement run, ambient air measurements were carried out on PM_10_, but only at the respective start and final stations. These concentrations were mostly in the range of 10 µg/m^3^ or below (see Supporting Information) and corresponded to the values from urban background stations of the Lower Saxony Air Monitoring Network (LÜN). However, there were bus and train platforms with significantly higher ambient air concentrations of PM_10_. For example, the values in the metro station at Hanover Central are consistently around 50 µg/m^3^ (see Supporting Information). At other stations in Brunswick and Hanover, smoking areas on the platforms, traffic or construction sites influenced the PM_10_ concentration in the passenger cabins when the doors were open. Therefore, the PM_10_ peaks do not always coincide with entry and exit of many people. An example is shown in Fig. 1C, where the highest PM_10_ concentrations were measured at a low occupancy density. In Fig. 2C, the sudden increase in PM_10_ is due to the boarding of a large number of people at the last station before Hanover Central. The WHO recommends for PM_10_ not to exceed a 24 h value of 45 µg/m^3^ ^37^. In the measurements carried out here, the mean values calculated from the starting point to the final station were mostly well below the WHO guideline. Only at BSVG, Bus Line 419 (Run 09) was a value of this order of magnitude measured at 41.1 µg/m^3^. However, some short-term peak values occurred in individual measurements.

The data sets shown in Fig. 3 are part of the complete concentration profile for the respective run, the time ranges are shown in the Supporting Information (Fig. S1). The decay curves can also be viewed as representative for other runs. Decay models of various complexity were tested, and the simplest model was selected on the basis of a residual analysis.

Figure 3A shows the decay behavior of PM_10_ in BSVG line 411 (Run 01). After closing the doors, the PM_10_ concentration drops to the ambient air level within 3–4 min. The decay curve follows the mono-exponential time law shown in Eq. (1) very precisely.

1 $$C\left( t \right) = A \cdot e^{{ - k_{1} \cdot t}} + C_{0}$$

C(t) is the concentration in the passenger cabin at time t, A is the initial maximum concentration, k_1_ is the decay constant and C_0_ is the background concentration. Figure 3B shows another scenario for the ÜSTRA line 4 (Run 19). It is obvious that if the doors are opened frequently the PM_10_ concentration hardly decreases. Only after the doors have been closed the PM_10_ concentration follows the single exponential decay law. The decay constants are k_1_ = 37.7 h^−1^ for Run 01 and k_1_ = 28.8 h^−1^ for Run 19. These values can be explained by the high air exchange in the passenger cabins, the turbulent transport and the adherence of the particles to surfaces, especially people. In comparison, gravimetric deposition is slow. A spherical particle with a diameter of 10 µm needs approx. 5 min to sink to the ground from a height of 1 m^38^.

### Sensors

The evaluation of the results so far has shown that carbon dioxide is primarily suitable as an indicator for the air quality in transport cabins when there is a high density of people, as this parameter is directly linked to human breathing and therefore also to other components of the breathing gas. In this respect, a low CO_2_ value usually also means good air quality. PM_10_, on the other hand, is linked to personal dynamics. Other parameters, such as the TVOC value (total volatile organic compounds), often measured by photoionization^39^, are not expedient here.

Today there are many types of gas and particle sensors available for monitoring indoor air quality, which work reliably and are space-saving at the same time^40–43^. The mapping of the spatial distribution of air pollutants has proven to be a successful tool for investigating health risks in road traffic^44^. Therefore, in the course of the project, the idea came up of constructing sensors with which the concentration of the respective target parameter can be tracked not only in terms of time but also in terms of location. This provides the operator with valuable information about the number of people at certain times and stops along a route.

**Figure 4:**
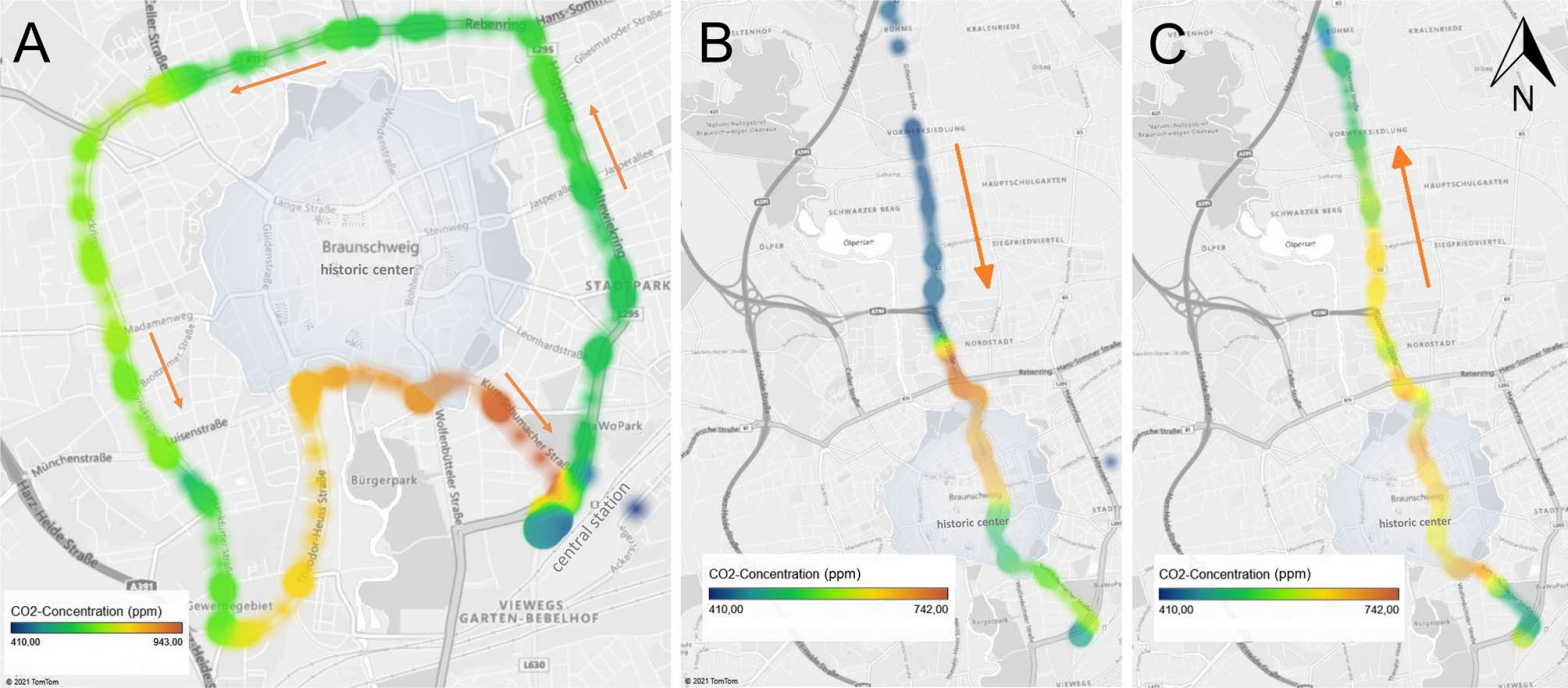
Contour plots of the carbon dioxide concentrations measured with gas sensors in buses. (**A**) BSVG Circle Line 419 (Run 09); (**B**) BSVG Tram Line 10 (Run 06, north to south); (**C**) BSVG Tram Line 10 (Run 05, south to north). The arrows indicate the direction of travel. The figure was generated with Microsoft Excel 2019 (v1808) using Microsoft Bing Maps and graphically modified with Microsoft PowerPoint 2019 (v1808).

Figure 4A shows the data from the carbon dioxide sensor along the BSVG Circle Line 419 (Run 09) as a contour plot. This line circles the Brunswick inner city. The sections of the route with an increased number of people are clearly marked by the red areas. BSVG Tram Line 10 shown in Figs. 4B,C passes through Brunswick City from north to south (Run 06) and from south to north (Run 05). Here, the highest occupancy (red areas) can be seen in the city center as expected.

**Figure 5:**
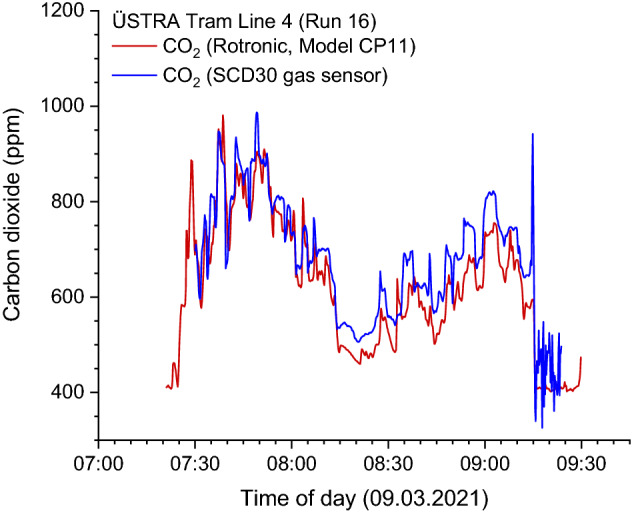
Comparison of measured carbon dioxide concentrations for the Rotronic CP11 and the SCD30 gas sensor. Both devices were calibrated versus the carbon dioxide concentration in ambient air.

The parameters PM_1_ and PM_2.5_, which were also measured with the sensors, did not provide any additional information for the issue of efficient ventilation, which is relevant here. There were also some deviations from the OPC data, which is due to the measurement technology. In the case of scattered light methods, it is not the size of the individually observed particle that is specified, but the size of a unit particle with the corresponding properties^31^. For example, the short-term PM_10_ peaks could not be recorded with the sensor. Moreover, the calibration of particle spectrometers is not traceable. This is different with carbon dioxide, since the sensors can be compared and adjusted with precisely calibrated devices. The comparison between the Rotronic CP11 and the sensor is shown in Fig. 5 for the carbon dioxide concentration at Run 16. Both devices were calibrated against the CO_2_ ambient air concentration. The differences in the measurement signals are always less than 10%, which is completely sufficient for this application.

## Conclusion

The investigations carried out here and the results presented do not claim to assess the risk of persons for an infection by pathogenic bioaerosols in passenger cabins in public transport. It is also explicitly warned against using this methodology for such purposes. Measured values are to be interpreted in such a way that with high occupancy, a low CO_2_ concentration implies a high air exchange, which significantly reduces the risk of infection. Conversely, an increased CO_2_ concentration indicates an increased risk of infection. It is recommended to choose a CO_2_ concentration of 1000 ppm as a criterion^33^, as this value has proven to be suitable for school classes^45^. Querol et al. come to analogous statements and conclude that with an air supply of 14 l/s/p an average CO_2_ concentration of 800–900 ppm should not be exceeded. The air supply should also be set in such a way that the basic climatic parameters temperature and relative humidity offer a comfortable thermal environment for the passengers.

However, it was demonstrated that the online measurement of simple parameters is a valuable tool for assessing air quality as a function of time, location and number of people. Previous studies have already shown that when occupancy is high, a low carbon dioxide concentration in particular indicates good ventilation, which automatically reduces the risk of infection. It is therefore recommended to take even more advantage of carbon dioxide as a control variable for air conditioning systems in transport cabins. In order to keep the risk of infection as low as possible, a value of 1000 ppm CO_2_ should not be exceeded even with high occupancy. Querol et al.^17^ suggest tracking the number of people online as well. This allows a person-related optimization of the ventilation conditions while saving fuel or electrical energy at the same time. The measurement can be carried out with low-cost sensors that are permanently installed in the cabin. The additional equipment with GPS also allows further evaluations of the dynamics of public transport.

## Supplementary Information

Supplementary Information.

## References

[1] Troko J (2011). Is public transport a risk factor for acute respiratory infection?. BMC Infect. Dis..

[2] Luo K (2020). Transmission of SARS-CoV-2 in public transportation vehicles: A case study in Hunan province. China. Open Forum Infect. Dis..

[3] Shen Y (2020). Community outbreak investigation of SARS-CoV-2 transmission among bus riders in Eastern China. JAMA Intern. Med..

[4] Pestre V (2012). Transmission by super-spreading event of pandemic A/H1N1 2009 influenza during road and train travel. Scand. J. Infect. Dis..

[5] Di Carlo P (2020). Air and surface measurements of SARS-CoV-2 inside a bus during normal operation. PLoS ONE.

[6] Kissler SM (2020). Reductions in commuting mobility correlate with geographic differences in SARS-CoV-2 prevalence in New York City. Nat. Commun..

[7] Hu M (2021). Risk of Coronavirus Disease 2019 Transmission in train passengers: An epidemiological and modeling study. Clin. Infect. Dis..

[8] Cui F (2011). Transmission of pandemic influenza A (H1N1) virus in a train in China. J. Epidemiol..

[9] Furuya H (2007). Risk of transmission of airborne infection during train commute based on mathematical model. Environ. Health Prev. Med..

[10] Moreno T (2021). Tracing surface and airborne SARS-CoV-2 RNA inside public buses and subway trains. Environ. Int..

[11] Rocklöv J, Sjödin H, Wilder-Smith A (2020). COVID-19 outbreak on the Diamond Princess cruise ship: Estimating the epidemic potential and effectiveness of public health countermeasures. J. Travel Med..

[12] Wagner BG, Coburn BJ, Blower S (2009). Calculating the potential for within-flight transmission of influenza A (H1N1). BMC Med..

[13] Lei H (2018). Routes of transmission of influenza A H1N1, SARS CoV, and norovirus in air cabin: Comparative analyses. Indoor Air.

[14] Lei H (2017). Logistic growth of a surface contamination network and its role in disease spread. Sci. Rep..

[15] Gupta JK, Lin C-H, Chen Q (2012). Risk assessment of airborne infectious diseases in aircraft cabins. Indoor Air.

[16] Gupta JK, Lin C-H, Chen Q (2011). Transport of expiratory droplets in an aircraft cabin. Indoor Air.

[17] Querol X (2022). How can ventilation be improved on public transportation buses? Insights from CO_2_ measurements. Environ. Res..

[18] Asadi S, Bouvier N, Wexler AS, Ristenpart WD (2020). The coronavirus pandemic and aerosols: Does COVID-19 transmit via expiratory particles?. Aerosol Sci. Technol..

[19] Morawska L, Cao J (2020). Airborne transmission of SARS-CoV-2: The world should face the reality. Environ. Int..

[20] Morawska L, Milton DK (2020). It is time to address airborne transmission of coronavirus disease 2019 (COVID-19). Clin. Infect. Dis..

[21] Browne A, St-Onge Ahmad S, Beck CR, Nguyen-Van-Tam JS (2016). The roles of transportation and transportation hubs in the propagation of influenza and coronaviruses: a systematic review. J. Travel Med..

[22] Casanova LM, Jeon S, Rutala WA, Weber DJ, Sobsey MD (2010). Effects of air temperature and relative humidity on coronavirus survival on surfaces. Appl. Environ. Microbiol..

[23] Lin K, Marr LC (2020). Humidity-dependent decay of viruses, but not bacteria, in aerosols and droplets follows disinfection kinetics. Environ. Sci. Technol..

[24] Yang W, Elankumaran S, Marr LC (2012). Relationship between humidity and influenza a viability in droplets and implications for influenza’s seasonality. PLoS ONE.

[25] Prussin AJ (2018). Survival of the enveloped virus phi6 in droplets as a function of relative humidity, absolute humidity, and temperature. Appl. Environ. Microbiol..

[26] Feng Y, Marchal T, Sperry T, Yi H (2020). Influence of wind and relative humidity on the social distancing effectiveness to prevent COVID-19 airborne transmission: A numerical study. J. Aerosol Sci..

[27] Salthammer T (2016). Children’s well-being at schools: Impact of climatic conditions and air pollution. Environ. Int..

[28] Morawska L (2013). Indoor aerosols: From personal exposure to risk assessment. Indoor Air.

[29] Morawska L (2017). Airborne particles in indoor environment of homes, schools, offices and aged care facilities: The main routes of exposure. Environ. Int..

[30] Boniardi L (2021). Commuting by car, public transport, and bike: Exposure assessment and estimation of the inhaled dose of multiple airborne pollutants. Atmos. Environ..

[31] Baron PA, Willeke K (2005). Aerosol Measurement. Principles, Techniques, and Applications.

[32] Parsons K (2002). Human Thermal Environments.

[33] Fromme H (2019). The German approach to regulate indoor air contaminants. Int. J. Hyg. Environ. Health.

[34] Persily A, de Jonge L (2017). Carbon dioxide generation rates for building occupants. Indoor Air.

[35] Kwon S-B, Youngmin C, Park D, Park E-Y (2008). Study on the indoor air quality of Seoul metropolitan subway during the rush hour. Indoor Built Environ..

[36] Licina D, Nazaroff WW (2018). Clothing as a transport vector for airborne particles: Chamber study. Indoor Air.

[37] World Health Organization. *WHO Global Air Quality Guidelines. Particulate Matter (PM2.5 and PM10), Ozone, Nitrogen Dioxide, Sulfur Dioxide and Carbon Monoxide*. (World Health Organization, 2021). 34662007

[38] Hinds WC (1999). Aerosol Technology.

[39] Zhang Y, Mo J, Salthammer T, Uhde E (2009). Real-time monitoring of indoor organic compound. Organic Indoor Air Pollutants.

[40] Kumar P (2015). The rise of low-cost sensing for managing air pollution in cities. Environ. Int..

[41] Kumar P (2016). Indoor air quality and energy management through real-time sensing in commercial buildings. Energy Build..

[42] Kumar P (2016). Real-time sensors for indoor air monitoring and challenges ahead in deploying them to urban buildings. Sci. Total Environ..

[43] deSouza P, Lu R, Kinney P, Zheng S (2021). Exposures to multiple air pollutants while commuting: Evidence from Zhengzhou. China Atmos. Environ..

[44] Kumar A, Mishra RK, Sarma K (2020). Mapping spatial distribution of traffic induced criteria pollutants and associated health risks using kriging interpolation tool in Delhi. J. Transp. Health.

[45] Birmili W, Selinka HC, Moriske HJ, Daniels A, Straff W (2021). Ventilation concepts in schools for the prevention of transmission of highly infectious viruses (SARS-CoV-2) by aerosols in indoor air. Bundesgesundheitsblatt.

