# Supplementary material for: Time and spatially resolved tracking of the air quality in local public transport
Source: Sci Rep. 2022 Feb 28;12:3262. doi: 10.1038/s41598-022-07290-5 (PMC8885640; doi:10.1038/s41598-022-07290-5)
Supplement: Supplementary file 1 — Supplementary Information. [file 41598_2022_7290_MOESM1_ESM.pdf]

SUPPORTING INFORMATION

Time and spatially resolved tracking of the air quality in local public transport

Tunga Salthammer[1,#], Christian Fauck[1], Alexander Omelan[1], Sebastian Wientzek[1], Erik Uhde[1]

[1]Fraunhofer WKI, Department of Material Analysis and Indoor Chemistry, Bienroder Weg 54 E, 38108 Braunschweig, Germany

[#]Corresponding author E-mail: tunga.salthammer@wki.fraunhofer.de

Table S1: Type designations of the vehicles in accordance with the information provided by the operating companies.

Operator	Route	Description
BSVG	Bus Line 411	Mercedes Benz Citaro C2 G Hybrid (2020), articulated bus, No. 2004 (Run 01) / 2001 (Run 02)
BSVG	Bus Line 419	Mercedes Benz O 530 Citaro Facelift G (2009), articulated bus, No. 0903
BSVG	Tram Line 3	Stadler Tramino Braunschweig Type II (2019), articulated railcar, No. 1953
BSVG	Tram Line 10	Alstom/LHB NGT8D, articulated railcar (2007), No. 0759
WestfalenBahn (WfB)	Line 95772<br>Line 95753<br>Line 95782<br>Line 95811	Stadler, Double-decker multiple unit “KISS”, six wagons
ÜSTRA	Tram Line 4	Alstom / Vossloh Kiepe, TW 3000 GTZ6-H, articulated railcar, two wagons, No 3133a
RegioBus	Regiobus 500	MAN Lion´s City A 20 (2018), No. H-RH 815

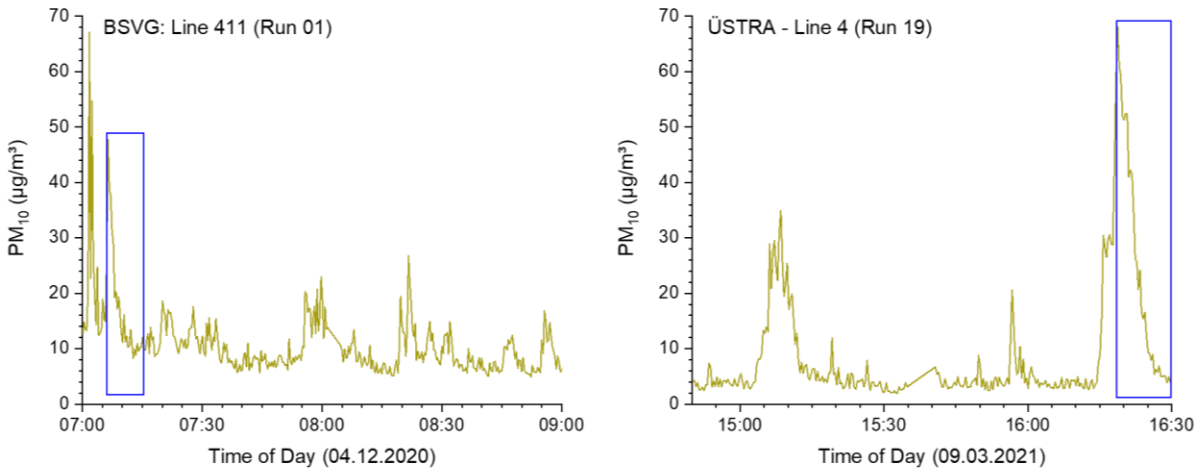

Figure S1: Measured PM_{10} concentrations for Run 01 and Run 19. The area outlined in blue denotes the data for the single exponential fits in Figure 3.

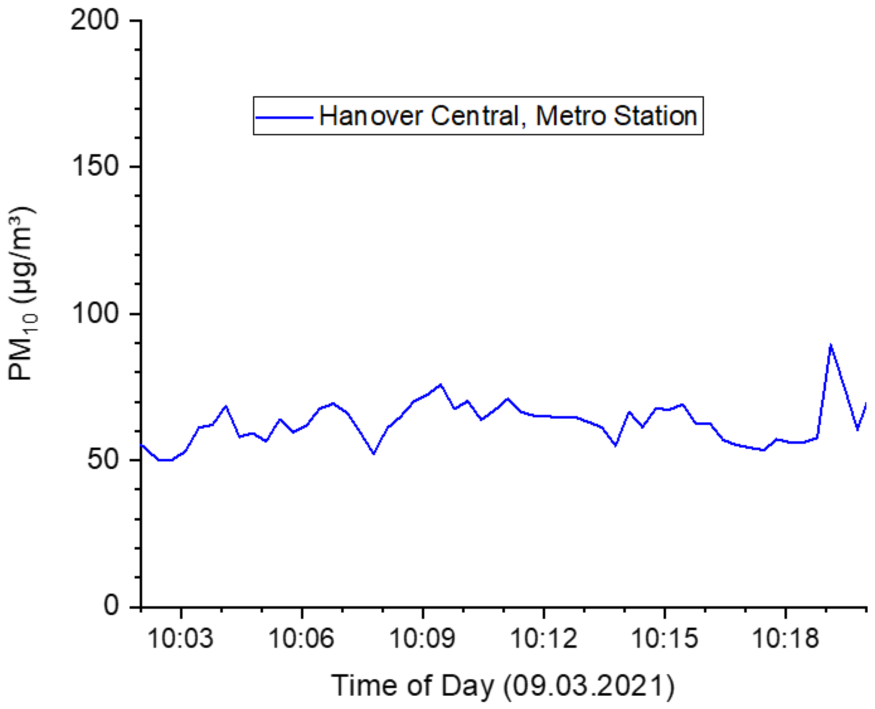

Figure S2: PM_{10} concentration in the Hanover Central underground station.

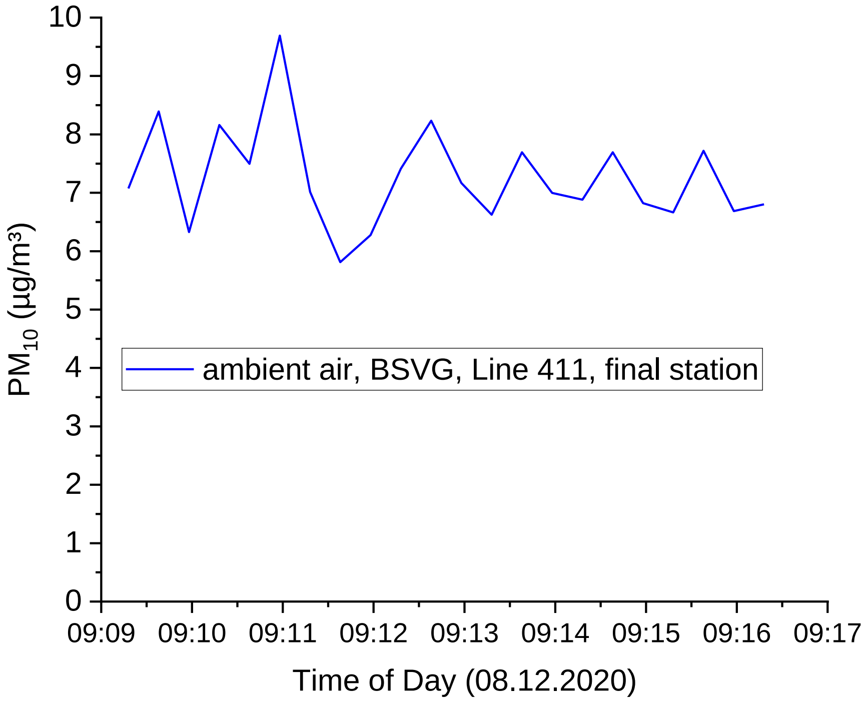

Figure S3: PM_{10} concentration in ambient air at the BSVG Line 411 final station.

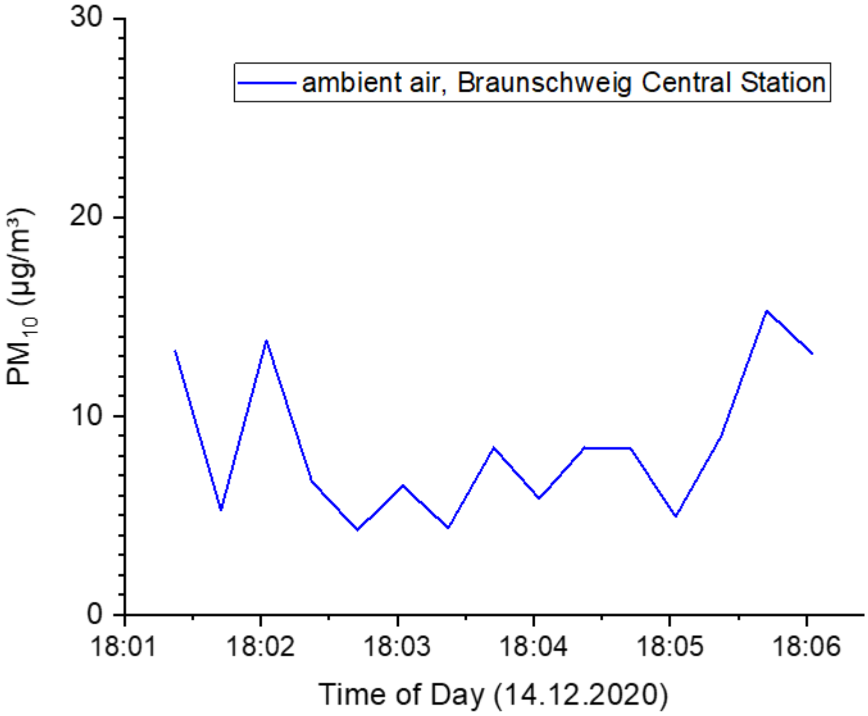

Figure S4: PM_{10} concentration in ambient air at the Brunswick Central Station.

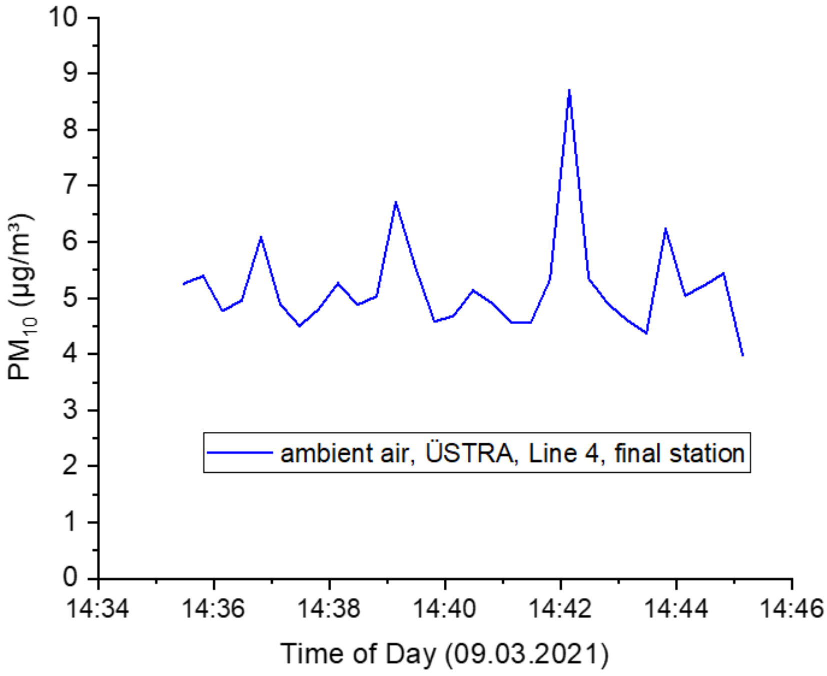

Figure S5: PM_{10} concentration in ambient air at ÜSTRA Line 4 final station.

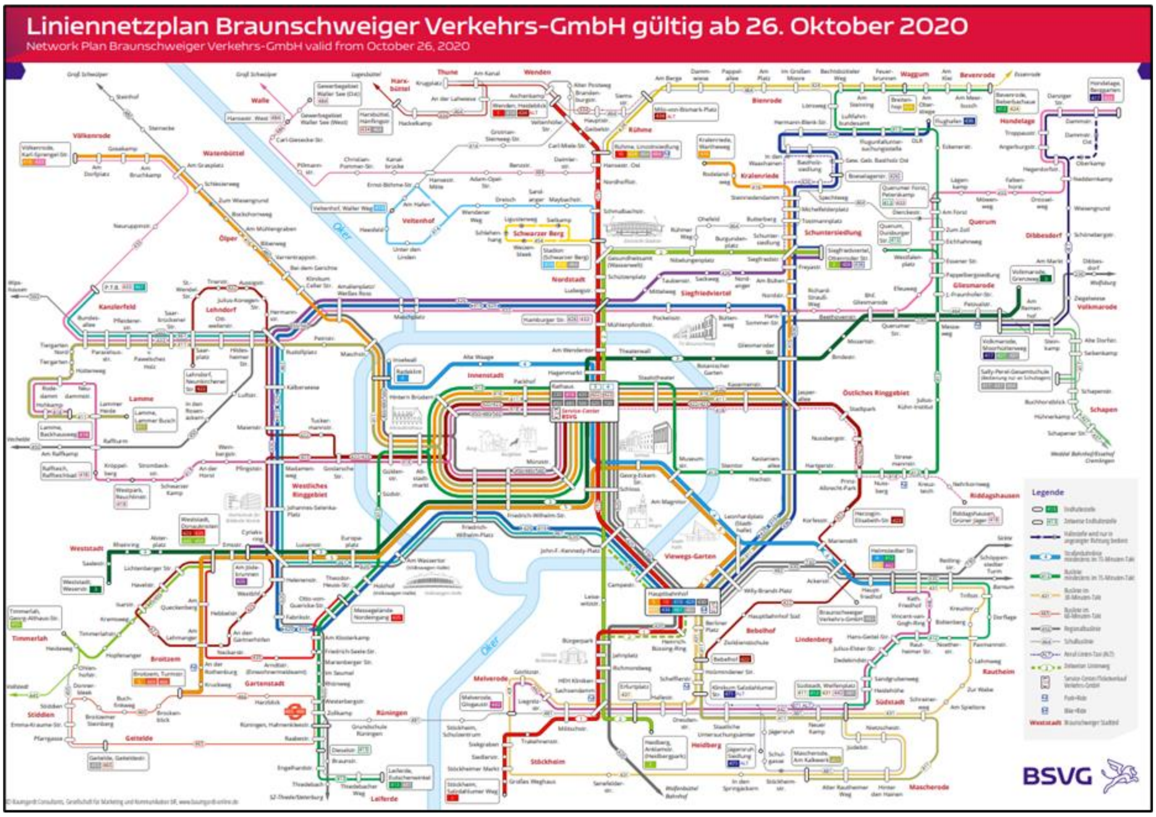

Figure S6: Route map of the BSVG (please zoom for a detailed view). Reprinted from https://www.bsvg.net with permission, all rights reserved.

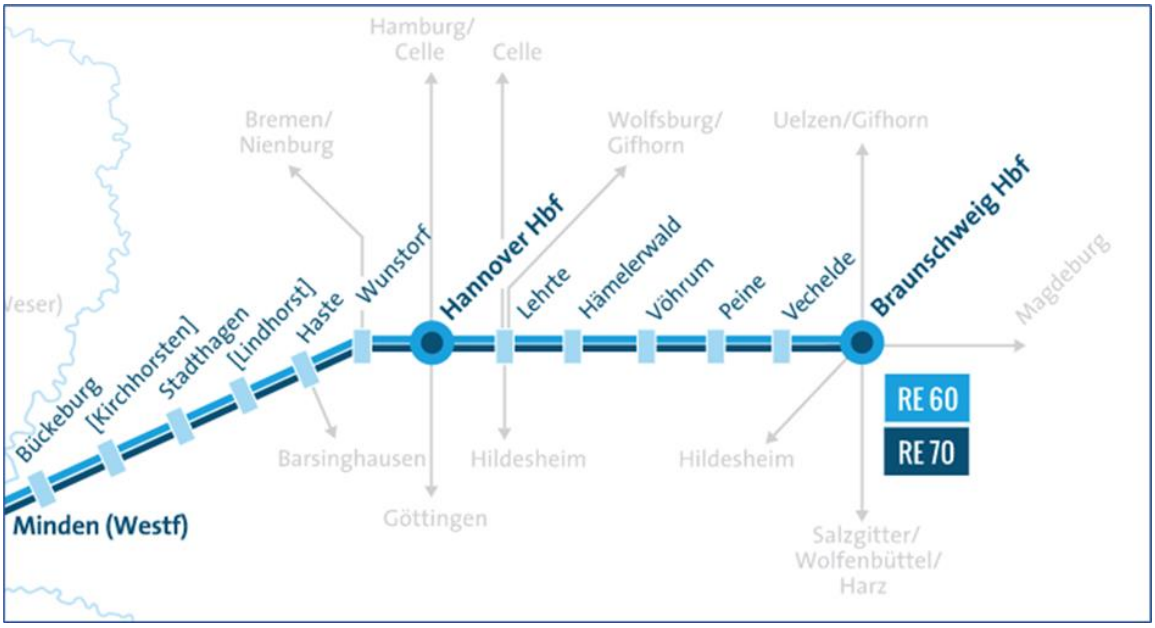

Figure S7: Route map of the WestfalenBahn (please zoom for a detailed view).). Reprinted from https://www.westfalenbahn.de with permission, all rights reserved.

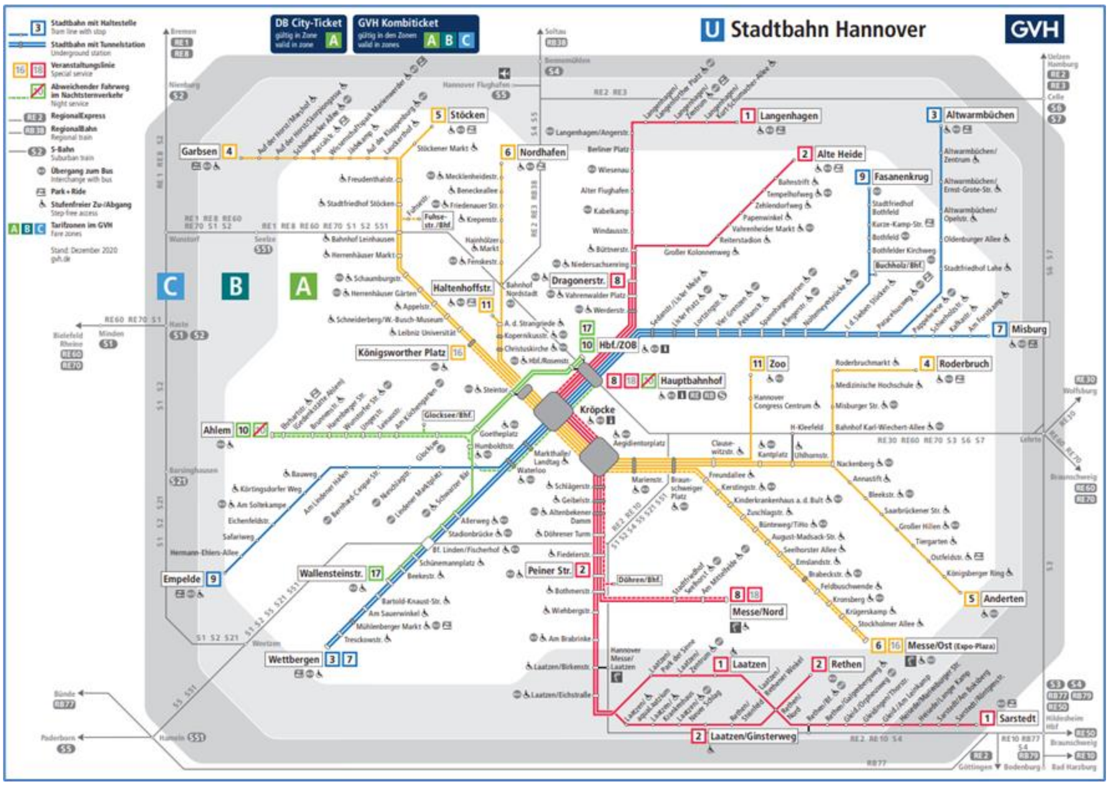

Figure S8: Route map of the ÜSTRA Stadtbahn (please zoom for a detailed view). Reprinted from https://www.uestra.de with permission, all rights reserved.

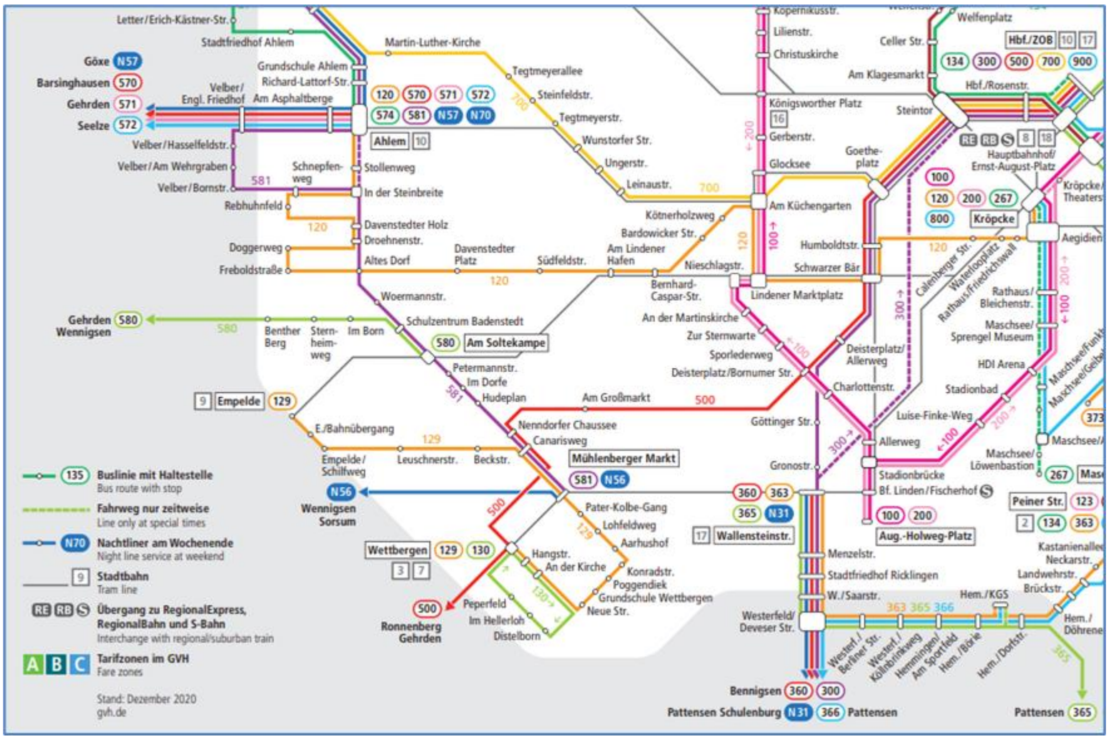

Figure S9: Route map of the Regiobus (please zoom for a detailed view). Reprinted from https://www.uestra.de with permission, all rights reserved.

Calculation of the carbon dioxide concentration in a closed space

$$C_{CO2}(t) = C_{CO2(ambient)} + 1000 \cdot \frac{N \cdot Q_{CO2}}{AER \cdot V} \cdot (1 - e^{-AER \cdot t})$$

$C_{CO2(ambient)}$ (ppm)	concentration of carbon dioxide in ambient air
N	number of persons in the room
Q_{CO2} (l/h)	carbon dioxide emission rate per person and hour
AER (h^{-1})	air exchange rate
V (m^3)	room volume
t (h)	time
